# Cemented versus non-cemented hemiarthroplasty of the hip as a treatment for a displaced femoral neck fracture: design of a randomised controlled trial

**DOI:** 10.1186/1471-2474-10-56

**Published:** 2009-05-28

**Authors:** Anne JH Vochteloo, DieuDonné Niesten, Roeland Riedijk, Willard J Rijnberg, Stefan BT Bolder, Sander Koëter, Keetie Kremers-van de Hei, Taco Gosens, Peter Pilot

**Affiliations:** 1 Department of Orthopaedics, Reinier de Graaf Gasthuis Delft, PO box 5011, 2600 GA, Delft, The Netherlands; 2 Department of Orthopaedics, Ziekenhuis Rijnstate Alysis Zorggroep Arnhem, PO box 9555, 6800 TA, Arnhem, The Netherlands; 3 Department of Orthopaedics, Canisius Wilhelmina Ziekenhuis Nijmegen, PO box 9015, 6500 GS, Nijmegen, The Netherlands; 4 Department of Orthopaedics, Elisabeth Ziekenhuis Tilburg, PO box 90151, 5000 LC, Tilburg, The Netherlands

## Abstract

**Background:**

A discussion is ongoing whether displaced femoral neck fractures in elderly patients should be treated with a non-cemented or a cemented hemiarthroplasty. A recent Cochrane analysis stresses the importance of further research into the relative merits of these techniques. We hypothesise that non-cemented hemiarthroplasty will result in at least the same technical-functional outcome and complication rate, with a shorter operation time.

**Methods and design:**

A randomised controlled multicentre trial will be performed.

The study population consists of 200 patients of 70 years and older. Patients with a displaced femoral neck fracture will be allocated randomly to have a cemented or a non-cemented hemiarthroplasty. Data will be collected preoperatively, immediately postoperatively, and 6 weeks, 3 months and 1 year postoperatively.

The main outcome measures of this study are technical-functional results of the hemiarthroplasty, duration of surgery, complications, and mid-thigh pain. Secondary outcome measures are living conditions at final follow up, self-reported health-related quality of life, and radiological evaluation of the hemiarthroplasty.

**Conclusion:**

A recent Cochrane analysis did not find arguments in favour of either non-cemented or cemented hemiarthroplasty. The forthcoming trial will compare treatment for a displaced femoral neck fracture by cemented versus non-cemented hemiarthroplasty. Our results will be published as soon as they become available.

**Trial Registration:**

Trial Registration Number NTR1508

## Background

Patients with a hip fracture form a heterogeneous group with a high mortality rate, frequently troubled with multiple and severe co-morbidities. They are usually frail and elderly, with more than 30% aged ≥ 85 years. In European series, hip fracture patients have a 30-day mortality of >10%, and a 1-year mortality of 25–30%.[1] Despite the advances in surgical and anaesthetic techniques during the last 20 years there has been no decrease in mortality after surgical repair of hip fractures. [1]

The discussion about cemented or non-cemented hemiarthroplasty is similar to the discussion about cemented or cementless prostheses in primary hip arthroplasty. With respect to the latter, conflicting data are presented by the Swedish and Norwegian arthroplasty registers. [2,3] The general opinion is that cementless hip arthroplasties are more suitable for younger patients owing to the quality of their bone stock. In hemiarthroplasty for hip fracture surgery there are insufficient data from randomised trials to conclude on the superiority of either type of arthroplasty. Some authors found patients with cemented stems to have less pain and better mobility postoperatively than those with an non-cemented press fit.[4] Mid-thigh pain is frequently reported in primary cementless total hip arthroplasties. However, the reported incidence varies tremendously; D'Lima et al.[5] in a control-matched study reported 34%~vs.~3%, while Grübl et al.[6] in a non-controlled series reported only 3% for cementless primary total hip arthroplasties.

In cemented hemiarthroplasties polymethylmethacrylate bone cement is applied at the time of surgery, forming a solid bond between the prosthesis and the femoral bone; on the contrary, in cementless prosthesis the bonding between prosthesis and femur is dependent upon bony in-growth. Potential advantages of cementing are a less post-operative mid thigh pain, as the prosthesis is firmly fixed within the femur, and a reduced long-term revision rate from loosening of the prosthesis. Major side effects of cement are cardiac arrhythmias and cardio-respiratory collapse, which occasionally occur on application. These potentially fatal complications are caused either by embolism from marrow contents forced into the circulation or by a direct toxic effect of the cement. [7] Another major disadvantage of a cemented prosthesis is that revision arthroplasty will be more difficult.

The non-cemented hemiarthroplasty used in our study is the DB-10 (Biomet, Warsaw, USA), a straight collared stem designed to provide primary stability and to prevent migration. The primary stability of the stem, which has a narrow tip to guide the implant, is optimised by metaphyseal anchoring: intimate trabecular bone contact and optimised load transfer to the dynamic bone through to the posterolateral angle situated in the axis of the neck. Secondary stability (prevention of migration) is facilitated by optimizing bone in-growth surface through full hydroxyapatite coating on macro-structured titanium and grooves on A/P sides.

The cemented hemiarthroplasty used in our study is the Müller Straight Stem prosthesis (Zimmer, Warsaw, USA), developed with the advice of Professor M.E. Müller. It has now been in use reliably for many years; Ried et al. found a 15-year survival of 94%. [8] The fluted structure of the stem, with the two particularly marked longitudinal grooves AP in the stem axis, enables good cement adhesion. The small proximal collar serves to compress the cement, prevents the stem from sinking into the cement, and, together with the fine-blasted surface of the straight stem, achieves a stable anchorage of the implant.

Both prostheses have a unipolar head.

## Methods and design

### Study design

A single blinded randomised clinical multicentre trial will be conducted. Patients will be randomised to 2 groups of 100 in the operation theatre by a randomisation computer (IRIS^®^, SDS Medical, The Netherlands). The treatment will be either a cemented hemiarthroplasty or a non-cemented hemiarthroplasty. If complications occur during the procedure, the surgeon can change the procedure to ensure best medical practice.

We acknowledge the possibility that the patients might be able to tell which prosthesis they have received due to seeing their X ray during the outpatient clinic visits.

All patients with a displaced femoral neck fracture who are admitted to one of the participating hospitals and meet the inclusion criteria are asked to join the study. In case of (mental) incompetence of the patient his/her legal representative is consulted to obtain informed consent.

The study design, procedures and informed consent are approved by the Medical Ethical Committee of South West Netherlands.

Trial number Netherlands Trial Registry NTR 1508, 

### Study population

The study will be conducted at the Orthopaedic Departments of the Reinier de Graaf Gasthuis Delft, Ziekenhuis Rijnstate Alysis Zorggroep Arnhem, Canisius Wilhelmina Ziekenhuis Nijmegen and Elisabeth Ziekenhuis Tilburg.

Authors PP and AV will be responsible for the data and safety monitoring.

Inclusion criteria: patients aged = 70 years, with a displaced femoral neck fracture (Garden type III or IV) not older than 7~days are included.

Exclusion criteria: patients with a pathological fracture, a fracture older than 7 days or ASA-V classification are excluded.

Accrual is planned in the period from August 2008 to August 2009.

### Intervention

Patients will be receive either a cemented hemiarthroplasty, type Müller Straight Stem (Zimmer, Warsaw USA), or a non-cemented hemiarthroplasty, type DB-10 (Biomet, Warsaw USA). Every surgeon will use the same surgical approach for all implants – either straight lateral or posterolateral. The approach of choice is up to the surgeon's preference, as Parker's Cochrane analysis has shown that insufficient evidence is available for superiority of either approach. [9]

Anaesthesia, analgesia and postoperative physical therapy will be standardized in both groups.

### Study variables

Preoperatively, social demographic data, the ASA classification, Groningen Activity Restriction Scale (GARS, from which the Parker mobility score can be derived) and the SF-12 are obtained from each patient. [10,12,13]

The main outcome measures of this study are the functional results, complication rate and duration of surgery of the hemiarthroplasty.

### Primary outcome measures

• Duration of surgery is defined as skin-to-skin surgical time, measured in minutes.

• Complications, divided in major and minor complications, are recorded during the follow up period of 1 year. The complications are ranked in the modified Elixhauser mode, as described by Parvizi.[11]

• Functional outcome is measured by the Timed-Up-and-Go (TUG) score and the Groningen Activity Restriction Scale (GARS). [10,14]

• Postoperative mid-thigh pain is defined as pain explicit in the front and mid part of the femur, and scored on a 4-point ordinal scale (non/mild/moderate/severe).

### Secondary outcome measures

Living conditions at final follow up are measured in percentage of pre-fracture situation.

• Self reported health-related quality of life is measured by the SF 12. [13]

• Standard radiological evaluation of hemiarthroplasty and cement positioning and adequate size of the hemiarthroplasty measured on plain AP and axial X-rays of the operated hip. Adequate AP positioning is defined as less than 10 degrees varus or valgus. Adequate axial positioning is defined as 0–15 degrees anteversion.

### Follow up

The primary follow up for each patient will be 1 year. The first postoperative assessment will be in a clinical setting as the patient is still in the hospital. Assessments at 6, 12 and 52 weeks postoperatively will be made in the outpatient clinic, unless the patient is not able to visit the outpatient clinic; in that case questionnaires are mailed to the patient (possibly through contact with a relative) to obtain as much data as possible.

### Determination of sample size

#### (a) Duration of surgery

It is expected that a cemented hemiarthroplasty on average will take 12 min longer than a non-cemented hemiarthroplasty. Taking into account an estimated standard deviation of 12 min, a 1-year mortality of 25% and 10% of patients being lost to follow up or providing insufficient data we need a minimum of 46 patients per group.

#### (b) Functional outcome

Only for patients who are admitted from their own homes or assisted living facilities, functional outcome is measured by the Timed-Up-and-Go (TUG) score and the Groningen Activity Restriction Scale, and mid-thigh pain a non-inferiority design is used. [10,14]

• TUG performed; Giving the fact that at discharge with a cut-off point of over 30 seconds is significantly associated with falls. Less than 24 seconds is considered good functioning and has hardly any chance to fall. 42 seconds is considered poor functioning with a considerable chance to fall. [14] About 30% of the patients have a poor score at discharge. About 70% of all patients are admitted from their own homes or assisted living facilities. If non-inferiority is considered for TUG, a worsening of score from 30 to 42 is considered clinically relevant, with a SD of 10 sec., giving 16 patients a group. Corrected for the percentage admitted from home or assisted living facility (70%) and the drop out of 35% gives 34 patients a group

• GARS: The GARS data for this group of patients are insufficient to make valid calculations of statistical power. While data for multiple sclerosis patients and healthy older patients are available, there are none for patients with a hip fracture. An estimate is made based on preliminary data from a prospective survey in progress. As soon as sufficient data from this survey are available a final power calculation for GARS will be done.

• Mid thigh pain

Rather varying figures about mid-tight pain are reported in literature. D'Lima [5] reported 40 vs. 3% whereas v/d Wal et al. [15] reported 20 vs. 12% in non-tight proximal fit prosthesis. Therefore the average values of these two studies are taken.

π1 = 30%, π 2 = 7.5%, π = (30%+7.5%)/2 = 18.75%

n_1 _= n_2 _≥ 21* (0,1875*(1-0,1875))/(0,225)^2 ^= 63.2.

If the 25% 1-year mortality combined with expected 10% lost-to follow-up (or missing values) is taken into account this number is raised by 35%, then a total of 86 patients a group is needed. [16]

#### (c) Complications

The complication rate in this patient category is rather high, but heterogeneous. The rate of thrombo-embolic complications may depend on the type of intervention (cemented vs. non-cemented).

However, the incidence of deep vein thrombosis (DVT) and pulmonary embolism (PE) is such that an unachievable high number of patients is needed to acquire adequate power.

In conclusion, the total number of patients per group a set on 100, making a total of 200 patients is needed for this study.

### Statistical analysis

#### Primary outcome measures

The differences in operation time, mid-thigh pain, complications and TUG test will be analyzed using a Student's t-test, setting the level of significance at *p *< 0.05.

#### Secondary outcome measures

We will use the chi-squared test or Fisher's exact test for the binary variables. In cases of paired binary data (outcome variables) we will use the McNemar test and a paired t-test for mean differences. In the case of a statistically significant association (*p *< 0.05), we will use a logistic regression to model the probability/odds of an outcome.

The variables obtained from the clinical evaluation will be tabulated and analysed as mean, standard deviation, minimum and maximum values. All analyses will be performed using SPSS software (SPSS Inc., Chicago, IL, USA).

Kaplan Meijer survival analysis will be done after final follow up, using failure (reoperation) and mortality as endpoints.

## Discussion

In the treatment of displaced femoral neck fractures with hemiarthroplasty in elderly patients the use of bone cement is a controversial topic. A cemented hemiarthroplasty has been used in the majority of cases in most countries, but the non-cemented prosthesis is gaining popularity. Parker et al. [1] concluded that there is only limited evidence from randomised studies that cementing prosthesis in place may reduce the amount of post-operative pain and may lead to improved mobility. Furthermore no study has adequately addressed long-term outcome measures concerning this topic.

Cementing has potential physiologically adverse side effects. The major side effects cardiac arrhythmias and cardio-respiratory collapse, which occasionally occur upon application; these potentially fatal complications are caused either by embolism from marrow contents forced into the circulation or by a direct toxic effect of the cement. [7] Transesophageal echocardiography could be used to monitor emboli during surgery, but logistics prevent this in our centres, as most patients are operated outside regular working hours. Nevertheless, Pitto et al. [17] have already shown severe embolic events and intraoperative pulmonary impairment during fixation of the cemented femoral component in total hip arthroplasty, while fixation without cement clearly demonstrated a low risk of embolism. Clark et al. [18] found a transient but significant reduction in cardiac output and stroke volume for those receiving cement.

In non-cemented prostheses, bone quality is of importance; this is generally poor in elderly patients. LaPorte et al. [19] stated two relative contraindications for non-cemented total hip prosthesis: interference with bone in-growth and inability to achieve a congruent fit; both of these preclude establishment of rigid initial stability.

The purpose of this study is to compare treatment for displaced femoral neck fracture by cemented versus non-cemented hemiarthroplasty. We hypothesise that, relative to cemented hemiarthroplasty, non-cemented hemiarthroplasty will result in at least the same technical-functional outcome and complication rate, with a shorter operation time. The results of this study will be presented as soon as they become available.

## Competing interests

Author PP has received fundings of both Biomet and Zimmer. However none of these were related to this project or used prosthesis'.

## Authors' contributions

PP and DN originated the idea for the study, led its design and will supervise the project. AV and PP participated in the design of the study and in developing the protocol. AV and PP will coordinate the trial and are responsible for data acquisition. WR, TG, SK, SB and RR provided extra protocol information in order to expand the original study design from single-centre to multi-centre. AV, TG, SK, RR and KK are responsible for data collection at their respective locations. All authors have read and corrected draft versions of the manuscript and have approved the final manuscript.

## Pre-publication history

The pre-publication history for this paper can be accessed here:


